# Identification of the natural ligand of Bet v 1

**DOI:** 10.1186/2045-7022-4-S2-P3

**Published:** 2014-03-17

**Authors:** Christian Seutter von Loetzen, Thomas Hoffmann, Maximilian Hartl, Kristian Schweimer, Wilfried Schwab, Paul Rösch, Olivia Hartl-Spiegelhauer

**Affiliations:** 1 Universität Bayreuth, Lehrstuhl für Biopolymere, Bayreuth, Germany

## 

The major birch pollen allergen Bet v 1 is the main elicitor of airborne type I allergies and belongs to the family 10 of pathogenesis related proteins. Bet v 1 is the most extensively studied allergen and is well characterized on the biochemical and immunological level. However, its physiological function remained elusive. Here we present the identification of quercetin-3-O-sophoroside (Q3OS) as the natural ligand of Bet v 1. We isolated Q3OS bound to Bet v 1 from mature birch pollen and confirmed its binding by reconstitution of the Bet v 1:Q3OS complex. Fluorescence, UV/VIS as well as HSQC (heteronuclear single quantum coherence) titration experiments and the comparison with model compounds, such as quercetin, proved the specificity of Q3OS binding. The definition of the binding site by NMR together with a computational model give a more detailed understanding and shed light on the physiological function of Bet v 1. We postulate that the binding of Q3OS plays an important but unexplored role during the inflammatory response and the Bet v 1 recognition by IgE.

